# Uncovering NOTCH1 as a Promising Target in the Treatment of *MLL*-Rearranged Leukemia

**DOI:** 10.3390/ijms241914466

**Published:** 2023-09-23

**Authors:** Jacqueline Fischer, Estelle Erkner, Rahel Fitzel, Pia Radszuweit, Hildegard Keppeler, Fulya Korkmaz, Giovanni Roti, Claudia Lengerke, Dominik Schneidawind, Corina Schneidawind

**Affiliations:** 1 Department of Medicine II, University Hospital Tuebingen, Eberhard Karls University, 72076 Tuebingen, Germany; jacquelinemarie.fischer@med.uni-tuebingen.de (J.F.); dominik.schneidawind@usz.ch (D.S.); 2 Department of Medicine and Surgery, University of Parma, 43121 Parma, Italy; 3 Department of Medical Oncology and Hematology, University Hospital Zurich, 8091 Zurich, Switzerland

**Keywords:** *MLL*-rearranged leukemia, NOTCH1, CRISPR/Cas9, SERCA inhibitor, targeted therapy

## Abstract

*MLL* rearrangement (*MLL*r) is responsible for the development of acute leukemias with poor outcomes. Therefore, new therapeutic approaches are urgently needed. The NOTCH1 pathway plays a critical role in the pathogenesis of many cancers including acute leukemia. Using a CRISPR/Cas9 *MLL-AF4/-AF9* translocation model, the newly developed NOTCH1 inhibitor CAD204520 with less toxic side effects allowed us to unravel the impact of NOTCH1 as a pathogenic driver and potential therapeutic target in *MLL*r leukemia. RNA sequencing (RNA-seq) and RT-qPCR of our *MLL*r model and *MLL*r cell lines showed the NOTCH1 pathway was overexpressed and activated. Strikingly, we confirmed this elevated expression level in leukemia patients. We also demonstrated that CAD204520 treatment of *MLL*r cells significantly reduces *NOTCH1* and its target genes as well as NOTCH1 receptor expression. This was not observed with a comparable cytarabine treatment, indicating the specificity of the small molecule. Accordingly, treatment with CAD204520 resulted in dose-dependent reduced proliferation and viability, increased apoptosis, and the induction of cell cycle arrest via the downregulation of MLL and NOTCH1 target genes. In conclusion, our findings uncover the oncogenic relevance of the NOTCH1 pathway in *MLL*r leukemia. Its inhibition leads to specific anti-leukemic effects and paves the way for further evaluation in clinical settings.

## 1. Introduction

Rearrangements of the human Histone-lysine N-methyltransferase 2A or myeloid/lymphoid/mixed lineage leukemia genes (KMT2A/MLL) are associated with a subtype of de novo and therapy-related acute myeloid and lymphoblastic leukemia (AML; ALL) [1] and result in a particularly poor prognosis in the majority of patients [2,3]. The *KMT2A* gene fuses with over 130 known partner genes, with ALL-1 fused gene from chromosome 4 (*AF4*) and ALL-1 fused gene from chromosome 9 (*AF9*) being the most common [4]. The poor outcomes for patients usually arise from resistance or fast relapse after conventional chemotherapy, leading to high morbidity and mortality rates [5]. To improve patient prognosis, novel treatment strategies are urgently needed. Therefore, innovative molecular-guided therapies targeting the causative signaling pathways driving these diseases are currently the hallmark of modern medicine.

To study the effect of *MLL* rearrangements in leukemic cells, we employed a representative human *MLL-AF4* and *MLL-AF9* translocation model generated with CRISPR/Cas9. Both cellular models show unlimited in vitro growth potential and allow us to overcome the challenge of the rapid differentiation of patient cells in culture systems [6]. In our models, the fusion transcripts are expressed under the endogenous promoter, thereby naturally mimicking the patient’s actual disease. This allows us to investigate leukemogenesis and disease mechanisms associated with *MLL* rearrangements. It is already known that transcription factors such as ZNF521 play a crucial role in sustaining leukemogenesis by altering the gene landscape in *MLL*r leukemia [7]. However, further understanding is required to overcome resistance and relapse after chemotherapy. Therefore, our CRISPR/Cas9 *MLL-AF4*/-*AF9* model is valuable for uncovering disease mechanisms and conducting pharmacological studies with high translational characteristics [6,8,9,10].

In this study, the analysis of CRISPR/Cas9 *MLL-AF4* and *MLL-AF9* cells identified *NOTCH1* as a potential target gene that is significantly overexpressed and activated in contrast with respective non-mutated CD34+ hematopoietic stem and progenitor cells (HSPCs). Dysregulation of the NOTCH1 pathway is known in a variety of solid cancers and hematological malignancies, especially in T-cell acute lymphoblastic leukemia (T-ALL) [11,12,13], where the determination of *NOTCH1* is already recommended for clinical routine and characterization [1]. However, little is currently known about the relevance of the NOTCH1 pathway in AML [14]. The NOTCH receptor family is composed of heterodimeric transmembrane proteins, responsible for stem cell differentiation and cell fate determination [15,16]. In humans, four different NOTCH receptors and five corresponding ligands, Delta-like (DLL)1, DLL4, Jagged (JAG)1, and JAG2, are known [17]. The NOTCH1 pathway is activated when the ligand binds to the receptor via calcium (Ca^2+^)-dependent repeats [18]. This is followed by a series of protein cleavage steps mediated by ADAM10/17 metalloproteases and the presenilin–γ-secretase complex, which produces the NOTCH intracellular domain (NICD) [18]. The NICD is then released into the nucleus, where it acts as a transcription factor by activating downstream targets [18]. Interestingly, Cyclin-dependent kinase 1 (CDK1) is responsible for the stability of the NICD [19]. Depending on the cellular context, NOTCH1 signaling may have controversial functions and be both oncogenic and tumor-suppressive [20,21]. Previous efforts to inhibit this pathway with γ-secretase inhibitors (GSIs) have demonstrated clinical efficacy, particularly in combined treatment approaches, but are limited by inducing severe gastrointestinal side effects [22,23,24]. Due to the importance of this pathway, especially in leukemia, another type of drug targeting sarco-endoplasmic reticulum Ca^2+^-ATPase (SERCA) has been established [25]. However, the SERCA inhibitors were not well tolerated due to their specific cardiac Ca^2+^ toxicity [25]. Recently, the SERCA inhibitor CAD204520 with reduced off-target toxicity has been established, which allows us to investigate the relevance of the NOTCH1 pathway and its therapeutic consequences in *MLL*r leukemia [26].

In this study, a variety of assays demonstrated the successful inhibition of the NOTCH1 pathway by CAD204520, resulting in specific anti-cancer activity. This was achieved via the dose-dependent downregulation of the NOTCH1 receptor and a reduction in proliferation and viability, with a concomitant increase in apoptosis. Furthermore, we demonstrated the induction of cell cycle arrest, which is mechanistically mediated by the downregulation of *MLL* target genes and *CDK1*, a chemokine receptor known to promote cell growth and invasion and which is a stabilizer of the NICD and CXCR4 (C-X-C chemokine receptor type 4) [19,27]. Our study established a rationale for the further investigation of NOTCH1 inhibition in a clinical trial to improve the poor prognosis of *MLL*r patients.

## 2. Results

### 2.1. NOTCH1 as a Potential Target in MLL-AF4/-AF9-Rearranged Leukemia

**Figure 1:**
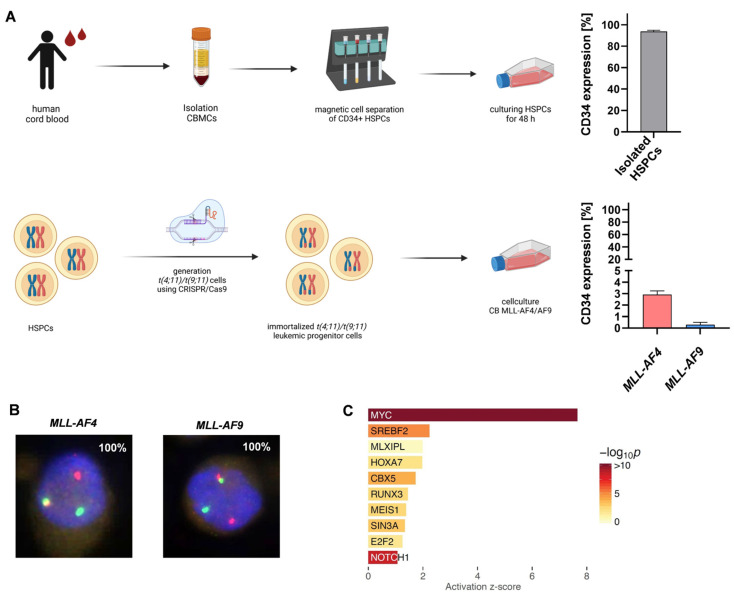

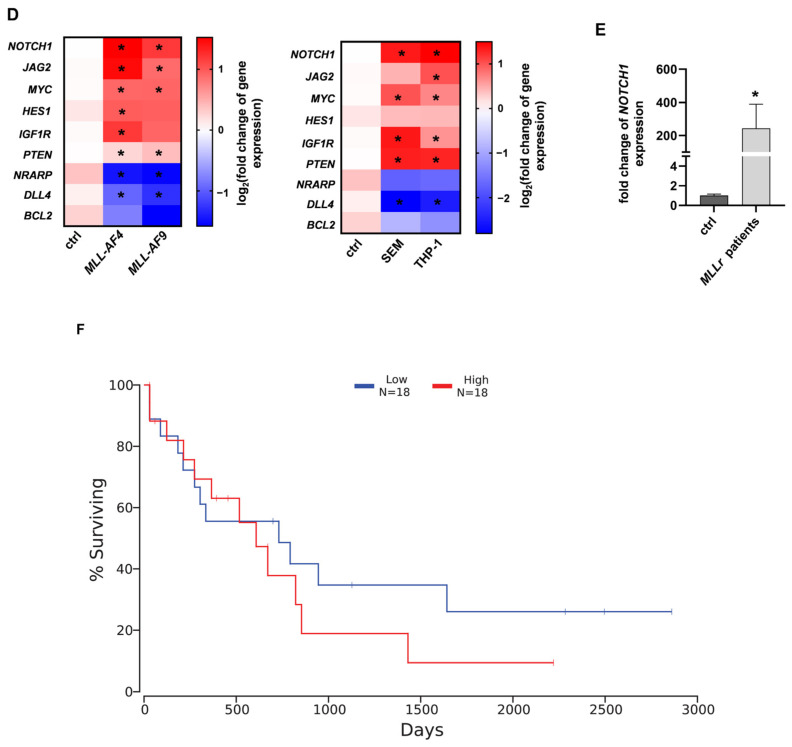
*NOTCH1* as a potential target gene in CRISPR/Cas9−induced *MLL−AF4/AF9*−rearranged leukemia cells: (**A**) CD34+ HSPCs were isolated from huCB via Ficoll separation and magnetic cell separation and cultured for 48 h. Purity of CD34+ cells (>90%) was measured using flow cytometry (*n* = 3). *t(4;11)*/*t(9;11)* was induced in cultured HSPCs using CRISPR/Cas9. After genome editing, generated *MLL-AF4* and *MLL-AF9* cells showed low CD34 expression (*MLL-AF4, n* = 3; *MLL-AF,9 n* = 3), measured using flow cytometry. (**B**) FISH analysis of CRISPR/Cas9 *MLL-AF4*/-*AF9* cells was performed after >30 days of culturing. Telomeric *MLL* 11q23.3 gene region is encoded by a red probe, centromeric *MLL* 11q23.3 gene region is encoded by a green probe. In CRISPR/Cas9 *MLL* rearranged cells, one *MLL* gene is rearranged (one green and one red signal) and one *MLL* gene is non-rearranged (one yellow signal). Manual inspection of 100 cells demonstrated a purity of 100%. (**C**) RNA sequencing of human CRISPR/Cas9 *MLL-AF4/AF9* cells (*n* = 2) compared with the respective control cells (huCB-derived CD34+, *n* = 2). Activation z-score indicates *NOTCH1* as a potential target. (**D**) Left heat map displays fold changes of *NOTCH1* and its associated genes in CRISPR/Cas9 *MLL-AF4/AF9* cells. Gene expression was measured using RT-qPCR (*MLL-AF4, n* = 3; *MLL-AF9, n* = 3) and normalized to huCB-derived CD34+ control cells (ctrl, *n* = 3). Right heat map display of fold changes of *NOTCH1* and its associated genes in *MLL-AF4* cell line SEM (*n* = 3) and *MLL-AF9* cell line THP-1 cells (*n* = 3). Gene expression was measured using RT-qPCR and normalized to huCB-derived CD34+ control cells (ctrl, *n* = 3). One-way ANOVA. * *p* < 0.05. (**E**) Fold change of *NOTCH1* overexpression in *MLLr* leukemia patients (*n* = 4, AML MLL-AF9), normalized to huCB-derived CD34+ control cells (ctrl, *n* = 3). Student’s *t*-test. * *p* < 0.05. (**F**) Kaplan–Meier survival curve (OncoLnc; 06/02/2023). Higher *NOTCH1* expression levels in AML patients show a trend toward worse survival rates. Log-rank *p* = 0.3.

Our previously established CRISPR/Cas9 *MLL-AF4/-AF9* model is an ideal platform to reveal potential therapeutic targets responsible for *MLL* leukemogenesis [8]. We therefore induced *t(4;11)*/*t(9;11)* by using CRISPR/Cas9 in CD34+ (> 90%) hematopoietic stem and progenitor cells (HSPCs) derived from human cord blood (huCB) (Figure 1A). The immortalized *MLL-AF4* and *MLL-AF9* cells were characterized by a patient typical myelomonocytic phenotype and low expression of CD34 (Figure 1A) [7]. After > 30 days of culturing, the purity of the rearranged cells was verified using fluorescence in situ hybridization (FISH) analysis in 100% of the cells (Figure 1B). Analysis of the RNA-sequencing (RNA-seq) data from these CRISPR/Cas9 *MLL-AF4/-AF9* cells identified a diverse set of upregulated genes that are associated with oncogenic potential. We used upstream regulator analysis and the activation z-score to identify changes in gene expression in *MLL*r leukemia cells compared with huCB-derived CD34+ HSPCs. Strikingly, this analysis revealed a positive z-score with a high probability (red color) for the *NOTCH1* gene, suggesting a potentially relevant role in disease maintenance (Figure 1C). To validate the RNA-seq results, we performed Reverse Transcription quantitative PCR (RT-qPCR) with the CRISPR/Cas9 *MLL-AF4* and *MLL-AF9* cells compared with the respective huCB-derived CD34+ control cells (nucleofected with Cas9 only), verifying *NOTCH1* overexpression in *MLL*r cells. Likewise, we also demonstrated the upregulation of the respective *NOTCH1*-associated target genes such as *HES1* (hairy and enhance of split-1), *IGF1R* (insulin-like growth factor receptor), and *PTEN* (phosphatase and tensin homolog), as well as the NOTCH1 ligand *JAG2* (Jagged-2). Importantly, *NRARP* (NOTCH regulated ankyrin repeat protein), a known suppressor of the NOTCH1 signaling pathway was significantly downregulated in *MLL*r cells, matching with the observed *NOTCH1* upregulation (Figure 1D) [28]. We confirmed this gene expression pattern in two commercially available *MLL*r cell lines as well, SEM and THP-1, using RT-qPCR (Figure 1D). To establish a connection between our model-based analysis and the clinic, we also analyzed patient samples with *MLL*r leukemia using RT-qPCR, which revealed significant *NOTCH1* overexpression compared with huCB-derived CD34+ cells (Figure 1E). Notably, regarding publicly available AML patient data, we assessed a trend toward improved overall survival with lower expression of NOTCH1 (Figure 1F). In summary, our data emphasize the potential relevance of the NOTCH1 pathway in *MLL*r leukemia.

### 2.2. The SERCA Inhibitor CAD204520 Specifically Affects the NOTCH1 Pathway

**Figure 2:**
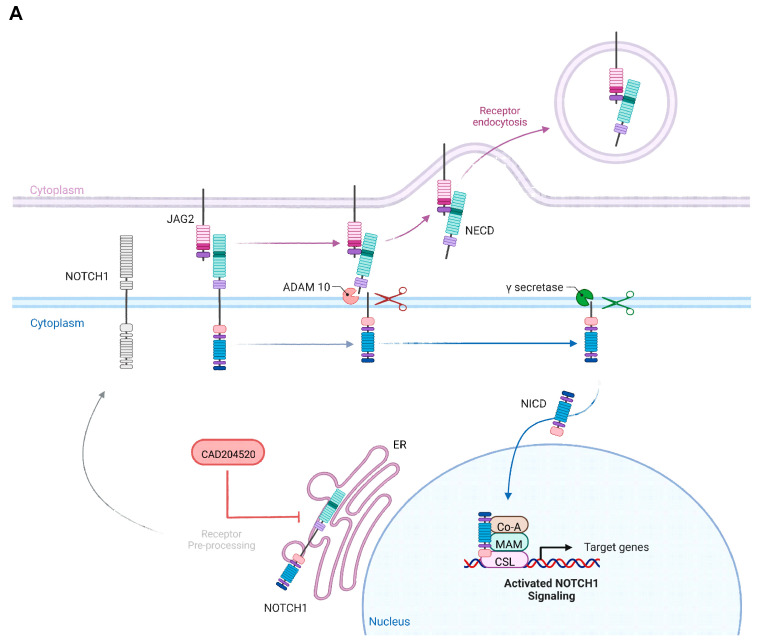

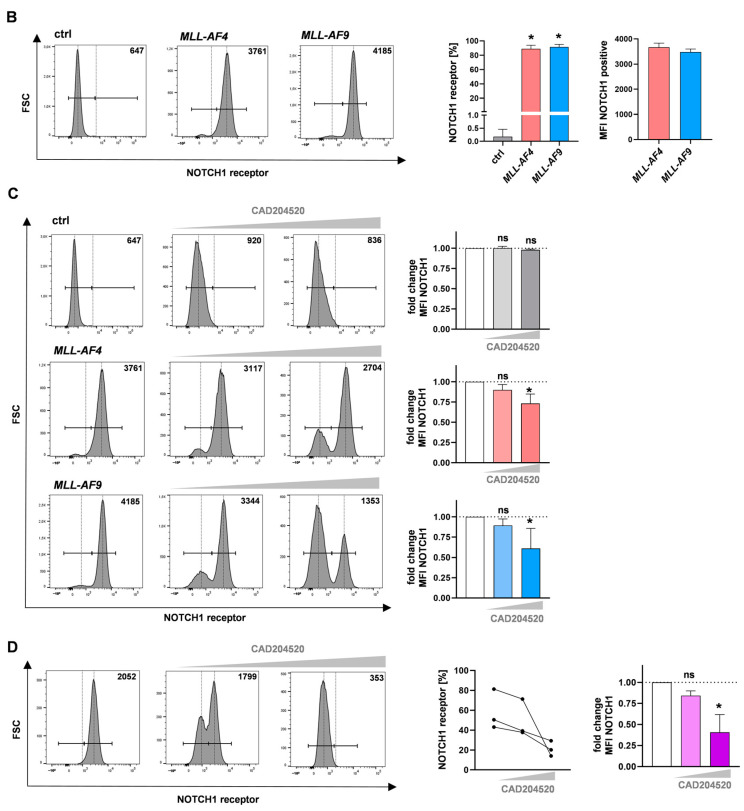
The SERCA inhibitor CAD204520 affects NOTCH1 receptor expression in MLL-AF4 and -AF9 cells. (**A**) Scheme of the NOTCH1 pathway and the mode of action of CAD204520. Created with BioRender. (**B**) Left—representative flow cytometry histograms of NOTCH1 receptor expression in CRISPR/Cas9 *MLL-AF4/-AF9* cells and huCB-derived CD34+ control cells (ctrl). Median fluorescence intensity (MFI) of NOTCH1-positive population was obtained with each single donor (CRISPR/Cas9 *MLL-AF4/-AF9* cells (*n* = 3/*n* = 3) and huCB-derived CD34+ control cells (*n* = 3, ctrl)), represented in flow cytometry histogram and as summarized data. In addition, NOTCH1 receptor expression percentage in CRISPR/Cas9 *MLL-AF4/-AF9* cells (*n* = 3/*n* = 3) and huCB-derived CD34+ control cells (*n* = 3, ctrl) is summarized. Significantly higher NOTCH1 expression in *MLL-AF4/-AF9* cells compared with ctrl. One-way ANOVA. * *p* < 0.05. (**C**) NOTCH1 receptor expression after 72 h CAD204520 treatment (4 µM; 8 µM) in CRISPR/Cas9 *MLL-AF4/-AF9* cells and huCB-derived CD34+ control cells (ctrl), normalized to vehicle control (DMSO), and analyzed using flow cytometry. Representative and summarized MFI of NOTCH1-positive population (*MLL-AF4, n* = 3; *MLL-AF9, n* = 3; ctrl, *n* = 3) shows a significant reduction in NOTCH1 receptor expression after inhibition in *MLL-AF4/-AF9* cells, which is not observed in ctrl. One-way ANOVA. * *p* < 0.05. not significant (ns) *p* > 0.05. (**D**) NOTCH1 receptor expression after 72 h CAD204520 treatment (4 µM; 8 µM) in *MLL*r primary patient cells (*n* = 3; acute myeloid leukemia (AML); *MLL-AF9*). Reduction in the NOTCH1 receptor (MFI of NOTCH1-positive population) normalized to vehicle control (DMSO) and analyzed using flow cytometry. One-way ANOVA. * *p* < 0.05. not significant (ns) *p* > 0.05.

To further evaluate the relevance of the observed overexpression of *NOTCH1* in our CRISPR/Cas9 *MLL-AF4/-AF9* cells as a potential target, we selected the recently developed SERCA inhibitor CAD204520 with a good side-effect profile as a promising NOTCH1 inhibitor in the following experiments [26]. CAD204520 carries out its mode of action by influencing the NOTCH1 receptor preprocessing in the endoplasmatic reticulum and hereby blocking the Ca^2+^-ATPase. Consequently, the lack of surface receptor expression leads to the interruption of the downstream signaling pathway (Figure 2A) [26]. Consistent with our transcriptomic findings, we detected significantly strong NOTCH1 receptor expression in the CRISPR/Cas9 model cells using flow cytometry, whereas NOTCH1 expression in the huCB-derived CD34+ cells was almost absent (Figure 2B). Importantly, treatment of the CRISPR/Cas9 *MLL-AF4* and *MLL-AF9* cells for 72 h with CAD204520 resulted in a significant dose-dependent reduction in NOTCH1 receptor expression, whereas huCB-derived CD34+ cells were not affected (Figure 2C). Furthermore, we also demonstrated a significant reduction in NOTCH1 receptor expression in primary cells derived from *MLL*r patients after the same CAD204520 treatment (Figure 2D).

**Figure 3:**
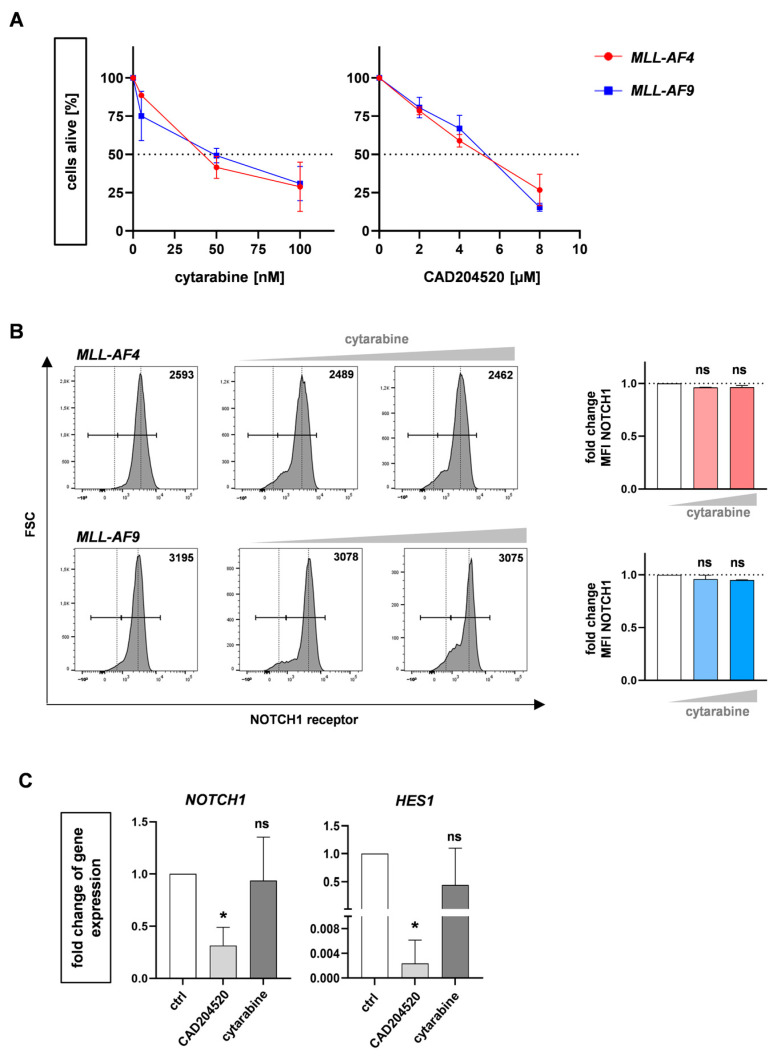
Comparable cytarabine treatment reveals specific effect of CAD204520 on the NOTCH1 pathway. (**A**) Viability of CRISPR/Cas9 *MLL-AF4/-AF9* cells treated with increasing dosage of CAD204520 or cytarabine for 72 h, measured using flow cytometry (eFlour506-) and normalized to their own vehicle control (DMSO/PBS) (*MLL-AF4, n* = 3; *MLL-AF9, n* = 3). (**B**) NOTCH1 receptor expression in CRISPR/Cas9 *MLL-AF4/-AF9* cells (*n* = 3) after 72 h cytarabine treatment (50 nM; 100 nM). NOTCH1 receptor expression (mean fluorescence intensity (MFI) of NOTCH1-positive population) normalized to vehicle control (PBS) is shown. One-way ANOVA. * *p* < 0.05. not significant (ns) *p* > 0.05. (**C**) Fold changes of *NOTCH1* and *HES1* in CRISPR/Cas9 *MLL-AF4/-AF9* cells (*n* = 3/*n* = 3) after 72 h CAD204520 (4 µM; 8 µM) or cytarabine (50 nM; 100 nM) treatment, normalized to their own vehicle control (DMSO/PBS). Significant reduction in gene expression after CAD204520, which is not observed after cytarabine treatment. Student’s *t*-test. * *p* < 0.05. not significant (ns) *p* > 0.05.

To further examine the specificity of CAD204520, we performed a comparable treatment with the commonly used AML chemotherapeutic agent cytarabine and CAD204520 to induce dose-dependent apoptosis in our CRISPR/Cas9 *MLL-AF4* and *MLL-AF9* cells (Figure 3A) and consequently assessed the NOTCH1 receptor and target gene expression using flow cytometry and qPCR, respectively. Using flow cytometry, we demonstrated that cytarabine did not induce a reduction in NOTCH1 receptors, unlike CAD204520, which reinforces CAD204520’s specificity toward *MLL*r leukemia cells via NOTCH1 (Figure 3B). Using RT-qPCR, we confirmed only CAD204520 influenced NOTCH1 and the downstream signaling pathway with a significant decrease in the NOTCH1-related gene HES1 known to be relevant in sustaining stem cell properties, whereas cytarabine had no influence (Figure 3C) [29]. In conclusion, our pharmaceutical studies demonstrate the specific inhibition of CAD204520 by influencing NOTCH1 expression and the downstream signaling pathway.

### 2.3. CAD204520 Induces Apoptosis and Cell Cycle Interruption Leading to Reduced Proliferation

**Figure 4:**
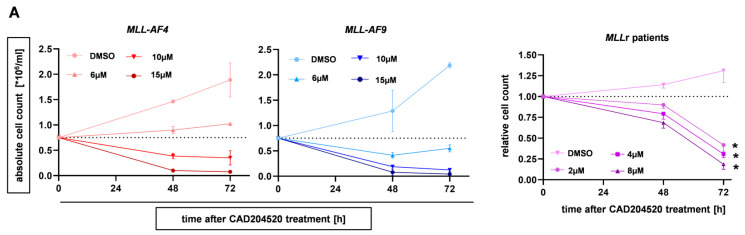

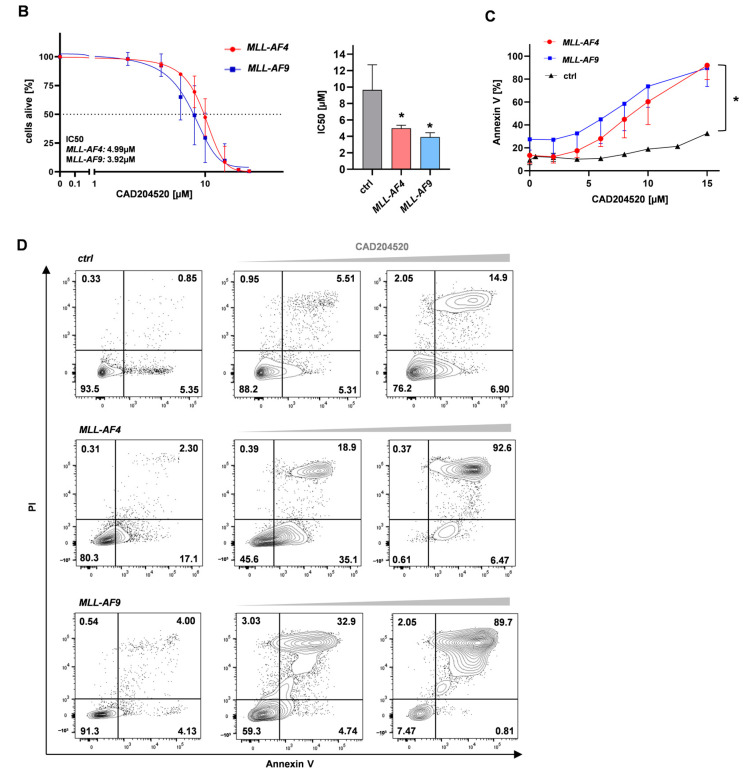
CAD204520 reduces proliferation in the *MLL*r model and primary leukemic cells and leads to apoptosis. (**A**) CRISPR/Cas9 *MLL-AF4/-AF9* (*n* = 2/*n* = 2) and *MLL*r patient cells (*n* = 3, AML *ML-AF9*) were treated with increasing concentrations of CAD204520 or vehicle control (DMSO) for 72 h. Live cells were counted after trypan blue staining in a Neubauer counting chamber. (**B**) CRISPR/Cas9 *MLL-AF4/-AF9* (*n* = 3/*n* = 3) and huCB-derived CD34+ control cells (ctrl, *n* = 3) were treated with increasing concentrations of CAD204520 or vehicle control (DMSO) for 72 h. Relative cell count was determined by counting cells in a Neubauer counting chamber after trypan blue staining. IC_50_ value of *MLL-AF4* was 4.99 µM, *MLL-AF9* 3.92 µM, and ctrl 9.78 µM. IC_50_ values of the dose-dependent curves were interpolated from a four-parameter logistic model. Significant difference between IC_50_ values of *MLL-AF4/-AF9* cells compared with those of ctrl. One-way ANOVA. * *p* < 0.05. (**C**) Annexin V staining, analyzed with flow cytometry, was used to determine the apoptotic effect of increasing CAD204520 treatment after 72 h incubation on CRISPR/Cas9 *MLL-AF4/-AF9* (*n* = 3, *n* = 3) and huCB-derived CD34+ control cells (ctrl, *n* = 3). Summarized data show a significant increase in apoptotic fraction (Annexin V+, PI−/+) in *MLL-AF4/-AF9* cells compared with ctrl. Student’s *t*-test. * *p* < 0.05. (**D**) Representative Annexin V staining histograms of CRISPR/Cas9 *MLL-AF4*/-*AF9* cells and huCB-derived CD34+ control cells (ctrl) after increasing CAD204520 treatment (DMSO; 6 µM, 10 µM). Percentages of counts in each population are presented.

To further investigate the leukemia inhibitory effect of CAD204520, we treated CRISPR/Cas9 *MLL-AF4* and *MLL-AF9* cells, as well as *MLL*r patient primary cells, for 72 h with different CAD204520 concentrations and assessed the dose-dependent inhibition of proliferation revealed with trypan blue staining and microscopy (Figure 4A). Accordingly, we generated the dose–response profiles of the *MLL-AF4* and *MLL-AF9* cells, respectively (Figure 4B). We revealed IC_50_ values of 4.99 µM for *MLL-AF4* and 3.92 µM for *MLL-AF9*, whereas huCB-derived CD34+ cells appeared more robust, with an IC_50_ of 9.65 µM (Figure 4B). To investigate the impact on cell apoptosis, we performed Annexin V staining with our CRISPR/Cas9 *MLL-AF4/-AF9* and huCB-derived CD34+ cells after treatment with CAD204520, measured using flow cytometry. In our models, we showed a dose-dependent increase in early (Annexin+, PI−) and late (Annexin+, PI+) apoptotic cells, whereas huCB-derived CD34+ control cells were significantly less influenced by identical CAD204520 treatment (Figure 4C,D).

**Figure 5:**
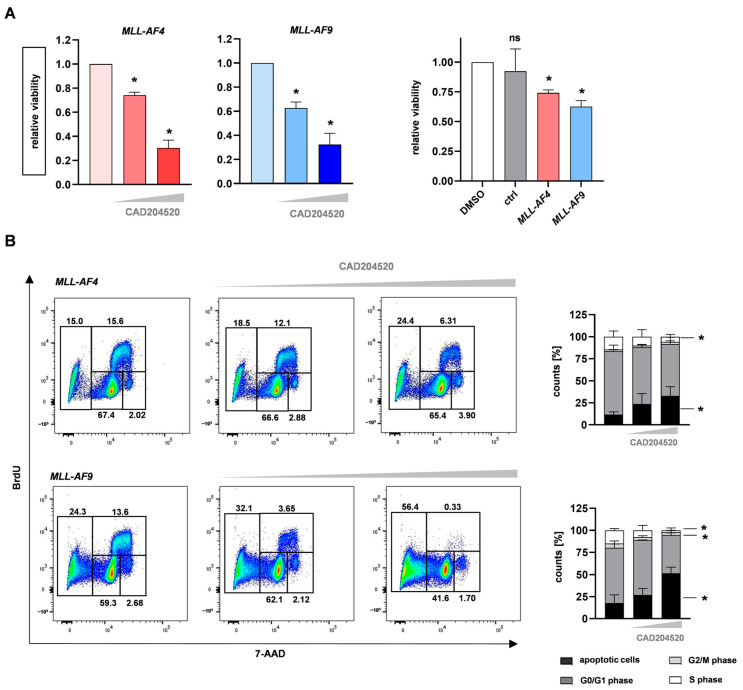
CAD204520 reduces cell viability and induces cell cycle interruption. (**A**) CAD204520 treatment for 72 h (DMSO; 6 µM, 10 µM) in CRISPR/Cas9 *MLL-AF4/-AF9* cells (*n* = 3/*n* = 3) decreased cell viability, measured using alamarBlue viability assay. huCB-derived CD34+ control cells (ctrl, *n* = 3) showed no significant reduction in viability compared with CRISPR/Cas9 *MLL-AF4/-AF9* cells (*n* = 3/*n* = 3) after 6 µM CAD204520 treatment. One-way ANOVA. * *p* < 0.05. not significant (ns) *p* > 0.05. (**B**) Representative (left) and pooled (right) data of BrdU cell cycle analysis of CRISPR/Cas9 *MLL-AF4/-AF9* cells (*n* = 3) after 72 h CAD204520 treatment (DMSO; 6 µM, 10 µM) show significant decrease in S-phase and G2/M-phase and increased apoptotic cells. One-way ANOVA. * *p* < 0.05.

Besides the impact of CAD204520 on cell proliferation and apoptosis, we further investigated the consequences of NOTCH1 inhibition on cell viability. Therefore, we treated our CRISPR/Cas9 *MLL-AF4* and *MLL-AF9* cells and huCB-derived CD34+ cells with selected CAD204520 concentrations or DMSO only (the vehicle control) for 72 h. Using the alamarBlue cell viability assay, we revealed dose-dependent and significantly reduced cellular viability in our model compared with the huCB-derived CD34+ control cells (Figure 5A). To figure out whether these anti-leukemic effects were accompanied by changes in the cell cycle, we performed bromodeoxyuridine (BrdU) and 7-amino-actinomycin D (7-AAD) staining, measured using flow cytometry. Interestingly, with increased concentrations of CAD204520, we assessed a significant decrease in the number of cells in the S and G2/M phases (Figure 5B). These data suggest that the inhibition of NOTCH1 results in convincing anti-leukemic effects via the reduction in proliferation and viability, induction of cell cycle arrest, and, finally, apoptosis in *MLL*-fusion-protein-driven leukemia, with significantly less impact on control cells.

### 2.4. Anti-Leukemic Effects of CAD204520 Are Linked to MLL Pathway Inhibition and Not to Cell Maturation

**Figure 6:**
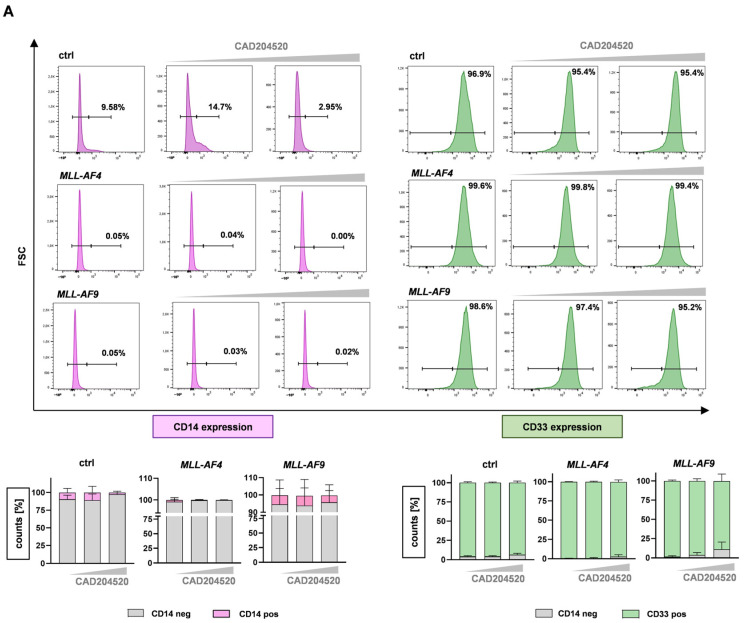

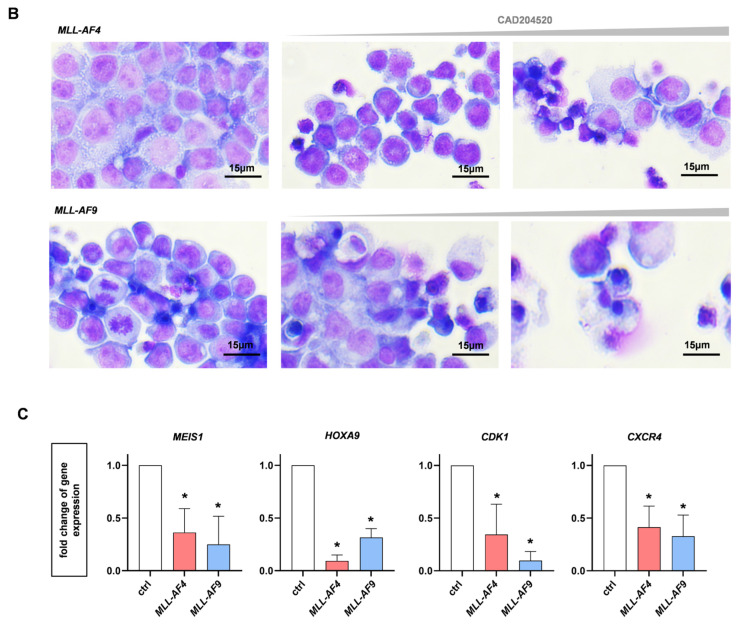
Anti-leukemic effects of CAD204520 are linked to MLL pathway inhibition and not to cell maturation. (**A**) Representative and pooled CD14 and CD33 expressions, measured using flow cytometry, as markers of differentiation. CRISPR/Cas9 *MLL-AF4/-AF9* (*n* = 3/*n* = 3) and huCB-derived CD34+ control cells (ctrl, *n* = 3) were treated with CAD204520 (6 µM, 10 µM) or vehicle control (DMSO) for 72 h. (**B**) Images show representative morphologies of CRISPR/Cas9 *MLL-AF4/-AF9* cells after 72 h CAD204520 treatment (DMSO; 6 µM, 10 µM). Pappenheim staining. Scale bar defines 15 µm. (**C**) Fold changes of *MEIS1*, *HOXA9*, *CDK1*, and *CXCR4* in CRISPR/Cas9 *MLL-AF4/-AF9* cells after 72 h CAD204520 treatment (8 µM), normalized to vehicle control (DMSO, ctrl), and measured using RT-qPCR with *n* = 3 different donors. Significant reduction in gene expression. One-way ANOVA. * *p* < 0.05.

*MLLr* leukemia cells are characterized by being arrested in an early stage of development [30]. We used CD14 as a marker of myeloid differentiation and CD33 as a marker for immature and aggressive leukemia [31] to characterize our CRISPR/Cas9 *MLL-AF4* and *MLL-AF9* cells before and following treatment with CAD204520. As expected, our patient-like *MLL*r model showed an immature immunophenotype with no CD14 and high CD33 surface expression using flow cytometry (Figure 6A). Likewise, huCB-derived CD34+ cells were CD14-negative and CD33-positive (Figure 6A). Following treatment, we did not detect an upregulation of CD14 or downregulation of CD33 in either *MLL*r cells or the huCB-derived CD34+ cells, indicating that the apoptotic effect was not induced by the maturation of the cells (Figure 6A). To substantiate this observation, we performed May–Gruenwald–Giemsa staining on CRISPR/Cas9-*MLL-AF4* and *MLL-AF9* cells after the CAD204520 treatment. Our translocated *MLL*r cells were characterized by a blastic morphology, a basophilic plasma, a huge nucleus with partially different shapes, and multiple nucleoli, consistent with patient leukemic cells (Figure 6B). After the inhibitor treatment, we could not detect any signs of maturation consistent with the observed immunophenotype, whereas only apoptotic characteristics such as a pyknotic nucleolus were visible (Figure 6B). As the anti-leukemic effects of CAD204520 were not induced by cell maturation, we measured the expression of *MLL* target genes after NOTCH1 inhibition with RT-qPCR as a hallmark of *MLL*r leukemogenesis. Interestingly, the *MLLr* target genes *MEIS1* and *HOXA9* were significantly downregulated in CRISPR/Cas9-*MLL-AF4* and *MLL-AF9* cells, indicating a mechanism responsible for apoptosis induced by CAD204520 (Figure 6C). Consistent with our findings concerning the impact of CAD204520 on the cell cycle, we observed a significant reduction in *CDK1*, which is known as a gene associated with the cell cycle and promotes tumor cell survival and resistance [19,32,33] (Figure 6C). Moreover, it has recently been discovered that CDK1 is essential for NICD stabilization and is therefore critical for NOTCH1 turnover [19]. Furthermore, after inhibitor treatment, we demonstrated a significant decrease in the expression of *CXCR4*, a NOTCH1-related gene, which is known to play a relevant role in the pathogenesis of ALL (Figure 6C) [34]. Our data suggest that CAD204520 does not manifest its anti-leukemic potential via cell maturation but via the downregulation of NOTCH1-related and *MLL*r target genes.

## 3. Discussion

In this study, we identified NOTCH1 as a driver of *MLL*r leukemogenesis, suggesting the potential to use this target as a molecular-guided treatment approach for *MLL*r leukemia. We used our innovative CRISPR/Cas9 *MLL*r model based on complete translocations of the *MLL* and *AF4* or *AF9* genes in HSPCs derived from huCB as an in vitro platform with unlimited growth potential. Using RNA-Seq and RT-qPCR, we demonstrated that *NOTCH1* and its respective target genes, such as *HES1* and *IGF1R*, are upregulated and highly activated in *MLL*r leukemia cells. Next to the oncogenic potential of *MLL* rearrangement via ZNF521 or EVI1, which enhances AML transformation [7], NOTCH1 upregulation is known to be particularly relevant in lymphoid leukemia such as T-ALL or chronic lymphocytic leukemia (CLL) and is associated with a poor prognosis [35,36,37]. As a target gene of NOTCH1, HES1 plays an essential role in the maintenance of T-ALL through the NOTCH–Hes1–CYLD–NFkB axis [38,39]. In contrast, it is known that HES1 mediates the tumor-suppressive roles of NOTCH1 signaling in AML development as well, which underlines the context-dependent manner of NOTCH1 signaling [40]. Moreover, IGF1R, also a NOTCH1 downstream target, is required for leukemia-initiating cell activity in T-ALL [41]. In addition to hematological diseases, other tumor entities with NOTCH1 activation have been identified, such as breast carcinoma [42]. Overall, this strengthens the importance of NOTCH1 and its pathway and creates a possibility to specifically target malignant cells via NOTCH1 inhibition.

Nevertheless, the majority of applied NOTCH1 inhibitors, such as GSIs or SERCA inhibitors, are associated with harmful effects that constrain clinical usage. GSI-related toxicity is based on a lack of substrate specificity resulting in a combined inhibition of mutated and wild-type NOTCH1, especially in intestinal progenitor cells [43]. Meanwhile, SERCA inhibition might cause cardiac toxicity due to calcium ion shift [44]. In contrast, the SERCA inhibitor CAD204520 demonstrates reduced Ca^2+^-related toxicity and yet retrains its anti-NOTCH1 effect, which made it an ideal compound for assessing the relevance of NOTCH1 in *MLL*r leukemia in this study [26]. In our study, we were able to demonstrate the specificity of this compound in blocking the NOTCH1 pathway by demonstrating a significant reduction in NOTCH1 receptor expression and target genes after CAD204520 treatment at the transcriptional and post-transcriptional levels using RT-qPCR and flow cytometry, whereas the widely used chemotherapy cytarabine showed no effect. Furthermore, we demonstrated the anti-leukemic impact of CAD204520 on *MLL*r cells by influencing cell proliferation, viability, and subsequent apoptosis, whereas huCB-derived CD34+ control cells were significantly less affected, allowing a therapeutic window in the application of CAD204520.

Concerning the potential mechanistic cell killing behind CAD204520 in *MLL*r cells, we demonstrated the cell cycle arrest and downregulation of *MLL* target genes upon inhibition, whereas their differentiation and morphology were not altered. This is consistent with the observation that the cell cycle is dysregulated in NOTCH1-mutated hematological malignancies, which mainly arise from the upregulation of responsible cell cycle genes of the cyclin-dependent kinase families [19]. Since CDK1 is also responsible for the stability of the NCID, we evaluated the *CDK1* expression after CAD204520 treatment and intriguingly uncovered its downregulation upon inhibition [19].

In addition to *CDK1*, *CXCR4* was also downregulated upon NOTCH1 inhibition. CXCR4 is a surface chemokine receptor and directs the leukemic cells in the bone marrow niche [45]. In T-ALL, oncogenic NOTCH1 activation is well-known to be related to enhanced CXCR4 expression [46]. This is also supported by observations in solid tumors, where NOTCH1 signaling was directly linked to elevated levels of CXCR4 [27]. Subsequently, by inhibiting NOTCH1, the repression of CXCR4 resulted in a reduction in tumor growth [27]. Importantly, CXCR4 has been proposed to support AML growth as well. However, to our knowledge, it has not been extensively studied in the context of elevated NOTCH1 expression [47]. Moreover, until now, only a few studies have assessed the pathogenic function of NOTCH1 as a prognostic marker or therapeutic target in AML [14]. In particular, even less is known about *MLL*r leukemia, a subtype of leukemia with a remarkably poor outcome [14,48,49,50].

Importantly, previous studies include a bioviability and tissue distribution study (PK study) of CAD204520 in CD1 mice [26]. The maximum concentration (c_max_) of 1.1 ng/mL (equivalent to 2.5 µM) falls within the range sufficient to cause an inhibitory effect in T-ALL [26] in vitro, indicating that an effect in preclinical AML models in vivo can also be achieved. While our in vitro study showed promising results in blocking NOTCH1 signaling using the therapeutic CAD204520, the use of monotherapies is unlikely to cure leukemia due to tumor heterogeneity and acquired resistance [26]. Therefore, considering the relationship of CDK and CXCR4 with the NOTCH1 pathway, one can argue that a combination of CAD204520 with compounds inhibiting either one of both mentioned pathways could further enhance the anti-leukemic efficiency. This is in line with other (clinical) studies showing promising results by inhibiting CXCR4 in AML or even proposing that the blockade of CDK family members, e.g., with palbociclib, could be a beneficial treatment option in combination with NOTCH1 inhibition for leukemia [2,35,51,52]. Therefore, our study not only uncovers the relevance of NOTCH1 in AML but also provides a convincing basis to further escalate the therapeutic targeting of NOTCH1 and combined treatment strategies in our model and clinical studies in the future to combat master oncogenic drivers in *MLL*r AML.

## 4. Materials and Methods

### 4.1. Human CRISPR/Cas9-MLLr Model

Human umbilical cord blood (huCB) was donated by the Center for Women’s Health (the Department of Gynecology) of the University Hospital Tuebingen (IRB approvals 751/2015BO2 and 461/2022BO2). Written consent was obtained from all patients in compliance with the Declaration of Helsinki. CD34+ hematopoietic stem and progenitor cells (HSPCs) were isolated from huCB via Ficoll separation and magnetic cell separation according to the manufacturer’s instructions (Miltenyi Biotech, Bergisch Gladbach, Germany) to obtain enrichment >90% determined using anti-CD34 flow cytometry analysis. CRISPR/Cas9 was used to induce *MLL-AF4* and *MLL-AF9* translocation as previously described [8]. *MLL*-rearranged (*MLL*r)-generated cells were cultured in StemMACS HSC Expansion Media XF (Miltenyi Biotec, Bergisch Gladbach, Germany) supplemented with 10% filtered fetal bovine serum (FBS; gibco by Thermo Fisher Scientific Inc., Waltham, MA, USA), 1% penicillin/streptomycin (Lonza, Basel, Switzerland), 50 ng/mL G-CSF (Chugai Pharmaceutical Co., Tokyo, Japan), FLT3-L, IL-3, IL-6, SCF, and TPO (PeproTech, Cranbury, NJ, USA), as well as 0,75 µM SR-1 and UM-729 (Stemcell Technologies, Vancouver, Canada). Purity was assessed via fluorescence in situ hybridization (FISH, Cytocell *MLL* (*KMT2A*) Breakapart Probe, Cambridge, UK) as described previously [8].

### 4.2. RNA Sequencing and Gene Expression Analysis

RNA was isolated (Machery Nagel NucleoSpin RNA Kit, Dueren, Germany) and quality was assessed with NanoDrop (Thermo Fisher Scientific Inc., Waltham, MA, USA) and Bioanalyser measurements (Agilent, Santa Clara, CA, USA). The RNA sequencing data quality was assessed using FastQC (V0.11.4, Babraham Institute, Cambridge, UK) [53] before aligning reads with STAR (V2.5.4b) [54] against the Ensembl H. sapiens genome V91. The alignment quality was analyzed using samtools (V1.1) [55]. For all genes, normalized read counts were obtained using GenomicAlignments (V1.14.2) and DESeq2 (V1.18.1) [56]. Transcripts covered with <50 reads were excluded from subsequent analyses. The significance thresholds were set to |log2 fold-change| ≥ 1 and BH-adjusted *p* ≤ 0.01. To minimize sample variations, surrogate variable analysis (sva, V3.26.0) was used [57]. nRPKMs (normalized reads per kilobase per million total reads) were calculated from raw counts from DESeq2 [58]. Using gene sets provided by Andersson et al. and Stam et al. [59,60], gene set enrichments were determined with GSEA (V3.0) [61,62]. RNA-seq data were deposited in NCBI’s Gene Expression Omnibus (GEO) and are accessible through GEO Series accession number GSE128342.

### 4.3. Cell Lines and Patient Samples

SEM cells (DSMZ ACC 546) were cultured in Iscove’s Modified Dulbecco’s Medium (IMDM) (Thermo Fisher Scientific, Waltham, MA, USA), FBS (gibco by Thermo Fisher Scientific Inc., Waltham, MA, USA), and 1% penicillin/streptomycin (Lonza Group AG, Basel, Switzerland). THP-1 cells (DSMZ ACC16) were cultured in Roswell Park Memorial (RPMI) 1620 medium (Thermo Fisher Scientific, Waltham, MA, USA) with 20% FBS (gibco by Thermo Fisher Scientific Inc., Waltham, MA, USA) and 1% penicillin/streptomycin (Lonza Group AG, Basel, Switzerland). *MLL*r patient peripheral blood mononuclear cells (PBMCs) were isolated from peripheral blood, received from and performed by the University Children’s Hospital Tuebingen. Written consent was obtained from all patients in compliance with the Declaration of Helsinki and approved by our Institutional Review Board (IRB approval 137/2017BO2). Primary patient cells were cultured in Roswell Park Memorial (RPMI) 1620 medium (Thermo Fisher Scientific, Waltham, MA, USA) with 10% FBS (gibco by Thermo Fisher Scientific Inc., Waltham, MA, USA) and 1% penicillin/streptomycin (Lonza Group AG, Basel, Switzerland).

### 4.4. Reverse Transcription Quantitative PCR (RT-qPCR)

RNA was isolated using the NucleoSpin RNA Kit according to the manufacturer’s instructions (Macherey-Nagel, Dueren, Germany). cDNA was generated with RevertAid H Minus Reverse Transcriptase, dNTP Mix, RiboLock RNase Inhibitor, and Random Hexamers (all Thermo Fisher Scientific, Waltham, MA, USA) according to the manufacturer’s protocol. RT-qPCR for the genes *NOTCH1*, *JAG2*, *MYC, HES1*, *IGF1R, PTEN*, *NRARP*, *DLL4*, *BCL2*, *CDK1*, *CXCR4*, *MEIS1*, *HOXA9*, and *18S* (Table 1, all Sigma-Aldrich, St. Louis, MO, USA) was performed using a Maxima SYBR Green qPCR Master Mix (Thermo Fisher Scientific, Waltham, MA, USA) according to the manufacturer’s protocol. A Maxima Probe qPCR Master Mix (Thermo Fisher Scientific, Waltham, MA, USA) was used for the amplification of the housekeeper *18S* rRNA (Sigma-Aldrich, St. Louis, MO, USA). Analysis was performed with a LightCycler 480 Instrument II (Roche Life Science, Penzberg, Germany). The fold change of gene expression was calculated according to the 2^−ΔΔCT^ method and normalized to 18S rRNA in relation to the respective control cells.

### 4.5. Inhibitor Treatment Assay

The NOTCH1 inhibitor CAD204520 was kindly provided by Marchesini et al. [26]. CAD204520 was diluted in DMSO at the concentrations used. Cytarabine (Stadapharm, Bad Vilbel, Germany) was diluted in PBS for the required working solutions. *MLLr* cells and huCB-derived CD34+ control cells were seeded with 7.5 × 10^5^ cells/mL. The inhibitors (CAD204520/cytarabine) were added to the cells and incubated for 72 h at 37 °C in the respective mediums described above.

### 4.6. Microscopy-Based Determination of Cell Counts

Total viable cell numbers were counted with 0.04% trypan blue (Sigma-Aldrich by Merck KGaA, Darmstadt, Germany) using a Neubauer counting chamber (Karl Hecht GmbH & Co. KG, Sondheim von der Rhön, Germany).

### 4.7. Cell Viability Assay

To determine cell viability, 10 µL of alamarBlue Cell Viability Reagent (Invitrogen, Waltham, MA, USA) was added to 90 µL of the cell suspension and incubated at 37 °C for 2 h according to the manufacturer’s protocol. The metabolized fluorochrome was detected with a Tecan Infinite M Plex Microplate Reader (Tecan, Maennedorf, Switzerland) at 560 nm.

### 4.8. Cell Cycle and Apoptosis Analysis

The Annexin V apoptosis assay and BrdU cell cycle analysis were performed using the FITC Annexin V Apoptosis Detection Kit I (BD Biosciences, Franklin Lakes, NJ, USA) and the FITC BrdU flow kit (BD Biosciences, Franklin Lakes, NJ, USA) according to the manufacturer’s protocol.

### 4.9. May–Gruenwald–Giemsa Staining

Cytospins were prepared by centrifuging 100 µL of cell suspension (4 min; 700 rpm; 21 °C) with a Shandon Cytospin 3 centrifuge (Thermo Fisher Scientific, Waltham, MA, USA) and stained with May–Gruenwald–Giemsa dye as previously described [63]. Images were taken using a Zeiss Primovert with an x40 objective and an Axiocam 105 color camera using ZEN software Version 3.0 blue edition (all Carl Zeiss AG, Oberkochen, Germany, https://www.zeiss.com/microscopy/de/produkte/software/zeiss-zen.html, accessed on 28 August 2023) at a resolution of 2560 × 1920 pixels.

### 4.10. Flow Cytometry

For the flow cytometric analysis of cell differentiation, cells were stained with anti-CD14 (BV785, clone M5E2, BioLegend, San Diego, CA, USA), anti-CD33 (Thermo Fisher Scientific, Waltham, MA, USA), anti-CD34 (Thermo Fisher Scientific, Waltham, MA, USA), and viability dye eFluor780 (eBioscience, San Diego, CA, USA).

Cells were stained with viability dye eFluor506 (eBioscience, San Diego, CA, USA), and intracellular NOTCH1 expression was detected with anti-NOTCH1 (PE, clone mN1A, BioLegend, San Diego, CA, USA) using the eBioscience Intracellular Fixation & Permeabilization Buffer Set (Thermo Fisher Scientific, Waltham, MA, USA) according to the manufacturer’s protocol. All measurements were performed with a BD LSRFortessa flow cytometer (BD Biosciences, Franklin Lakes, NJ, USA) and analyzed with FlowJo Version 10.8 (BD Biosciences, Franklin Lakes, NJ, USA).

### 4.11. Statistical Analysis

Statistical analysis was performed using one-way ANOVA or Student’s t-test as indicated in each figure legend. *p*-values of < 0.05 were considered statistically significant. The IC_50_ values of the dose–response curves were interpolated from a four-parameter logistic model as previously described [8]. All data were analyzed with Prism 7.03 (GraphPad Software, La Jolla, CA, USA).

## 5. Conclusions

In this study, we demonstrated the relevance of NOTCH1 in MLLr leukemia by using the NOTCH1 inhibitor CAD204520. By using our patient-like CRISPR/Cas9 *MLL-AF4*/-*AF9* model, we showed a significant influence on cell proliferation, viability, and subsequent apoptosis, whereas huCB-derived CD34+ control cells were less affected. Furthermore, we demonstrated a specific reduction in the NOTCH1 receptor and gene expression after inhibitor treatment as well as the downregulation of MLL and NOTCH1-related downstream targets like *HOXA9* or *CXCR4*. In conclusion, our findings uncover the oncogenic relevance of the NOTCH1 pathway in *MLL*r leukemia and provide a promising target in the treatment of *MLL*r leukemia.

## Figures and Tables

**Table 1:** Primer sequences used for RT-qPCR.

Gene	Forward Primer Sequence 5′-3′	Reverse Primer Sequence 5′-3′
***NOTCH1***	GCCTGGACAAGATCAATGAGTTC	TCCACATCGTACTGGCACAGA
***JAG2***	GACAACGATACCACCCCGAAT	CATGCGACACTCGCTCGAT
***MYC***	CCTGGTGCTCCATGAGGAGAC	CAGACTCTGACCTTTTGCCAGG
***HES1***	GGACATTCTGGAAATGACAGTGAA	CCCAGCACACTTGGGTCTG
***IGF1R***	GCCCCTCGGGCTTCAT	ACCTTCACAAGGGATGCAGTACA
***PTEN***	GGGAAGTAAGGACCAGAGACAAAAA	AGCGCCTCTGACTGGGAATA
***NRARP***	GCTGCACCAGTCGGTCATC	CCGAACTTGACCAGCAGCTT
***DLL4***	CCCTGGCAATGTACTTGTGATG	GAGTGGTGGGTGCAGTAGTTGAG
***BCL2***	CCTGTGGATGACTGAGTACCTGAAC	CAGCCAGGAGAAATCAAACAGA
***CDK1***	CCATTGACTAACTATGGAAGATTATACCA	TGTCTACCCTTATACACAACTCCATAGG
***CXCR4***	TGGAGGGGATCAGTATATACACTTCA	TCATAGTCCCCTGAGCCCATT
***MEIS1***	TGGCCACACGTCACACAGT	TTTGTCCTTATCAGGGTCATCATC
***HOXA9***	ATGAGAGCGGCGGAGACA	CGCGCATGAAGCCAGTT
***18S***	CGGCTACCACATCCAAGGAA	GCTGGAATTACCGCGGCT

## Data Availability

For the original data, please contact corina.schneidawind@usz.ch.
